# CicArVarDB: SNP and InDel database for advancing genetics research and breeding applications in chickpea

**DOI:** 10.1093/database/bav078

**Published:** 2015-08-18

**Authors:** Dadakhalandar Doddamani, Aamir W. Khan, Mohan A. V. S. K Katta, Gaurav Agarwal, Mahendar Thudi, Pradeep Ruperao, David Edwards, Rajeev K. Varshney

**Affiliations:** ^1^Research Program Grain Legumes, International Crops Research Institute for the Semi-Arid Tropics (ICRISAT), Hyderabad 502 324, Telangana State, India,; ^2^School of Agriculture and Food Sciences, University of Queensland, St Lucia, Queensland, Australia 4072,; ^3^School of Plant Biology, The University of Western Australia, Perth, Western Australia, Australia 6009 and; ^4^Institute of Agriculture, The University of Western Australia, Perth, Western Australia, Australia 6009

## Abstract

Molecular markers are valuable tools for breeders to help accelerate crop improvement. High throughput sequencing technologies facilitate the discovery of large-scale variations such as single nucleotide polymorphisms (SNPs) and simple sequence repeats (SSRs). Sequencing of chickpea genome along with re-sequencing of several chickpea lines has enabled the discovery of 4.4 million variations including SNPs and InDels. Here we report a repository of 1.9 million variations (SNPs and InDels) anchored on eight pseudomolecules in a custom database, referred as CicArVarDB that can be accessed at http://cicarvardb.icrisat.org/. It includes an easy interface for users to select variations around specific regions associated with quantitative trait loci, with embedded webBLAST search and JBrowse visualisation. We hope that this database will be immensely useful for the chickpea research community for both advancing genetics research as well as breeding applications for crop improvement.

**Database URL:** http://cicarvardb.icrisat.org.

## Introduction

Chickpea (*Cicer arietinum)* ranks second in production among grain legumes after common bean. It is a challenging task for breeders to increase crop production to meet the growing demand for this crop. Genetic and genomic resources such as molecular markers, genetic and physical maps and trait associated markers are valuable sources for breeders for crop improvement.

Significant efforts have been made by chickpea breeders and over 350 cultivars have been released so far, India being the largest grower of chickpea (1). Recently sequenced draft genome of *kabuli* chickpea (CDC frontier), along with whole genome re-sequencing (WGRS) data of 29 genotypes and 61 sequenced using restriction site-associated DNA (RAD) have provided genomic resources to support functional genomics and molecular breeding activities for chickpea improvement (2).

Single nucleotide polymorphisms (SNPs) are common genetic variants used to identify candidate genes and genotype–phenotype association studies (3). Most significant uses are estimation of breeding value of genotypes and marker-assisted selection studies (4). Concerted efforts of diverse research groups (3, 5–12) have led to the development of many SNP markers. Recent advances in DNA sequencing technologies such as next generation sequencing (NGS) facilitate the discovery of large numbers of SNPs at relatively low cost (13–15). Publicly available alignment algorithms and variant detection tools have facilitated mapping of short read sequences and detection of variations with respect to reference genome sequence (16).

A SNP database should help users access a wide variety of biological data by querying with simple commands in both an easy and comprehensive manner. Considering the significance of markers for crop improvement, several attempts have been made to develop marker repositories in various crops, e.g. rice (17, 18), maize (19), wheat (20), soybean (21, 22), barley (18, 23), brassica (18) etc. autoSNPdb (http://autosnpdb.appliedbioinformatics.com.au/) (18, 23) is generalised SNP database storing information for species namely rice, wheat, barley and brassica.

Legume information system (LIS) and Chickpea Genomic Web Resource (CGWR) mainly provides accessibility to the chickpea genome and its features through genome browsers and quantitative trait loci maps to the users. The current study brings together all publically available information on SNPs/InDels to the chickpea research community. A SSR marker repository entitled CicArMiSatDB (24) for chickpea was developed and now similar efforts have been initiated to develop a SNP database in chickpea to make the SNP data publically available mainly for assisting breeders in varietal improvement. In the past, several thousand SNPs were identified and reported using RNA-sequencing technologies (5, 8). With the availability of reference genome along with re-sequence data from 90 chickpea lines using WGRS or RAD sequencing, several million variants including SNPs have been identified (2). With an objective to enhance utilization of the identified SNPs/InDels for chickpea genetics and breeding applications, this study reports a database containing large-scale variants along with characterization in the genome.

## Material and methods

SNP and InDel variations were identified from WGRS of 29 varieties and RAD sequencing of 61 varieties (2). SNPs located on the pseudo-molecules were used to develop CicArVarDB. The SNPs located on the pseudomolecules were arranged as a binary matrix based on their presence/absence across the 90 genotypes. The final data set was stored in a relational database constructed using PostgreSQL (v9.2.4).

**Figure 1.**
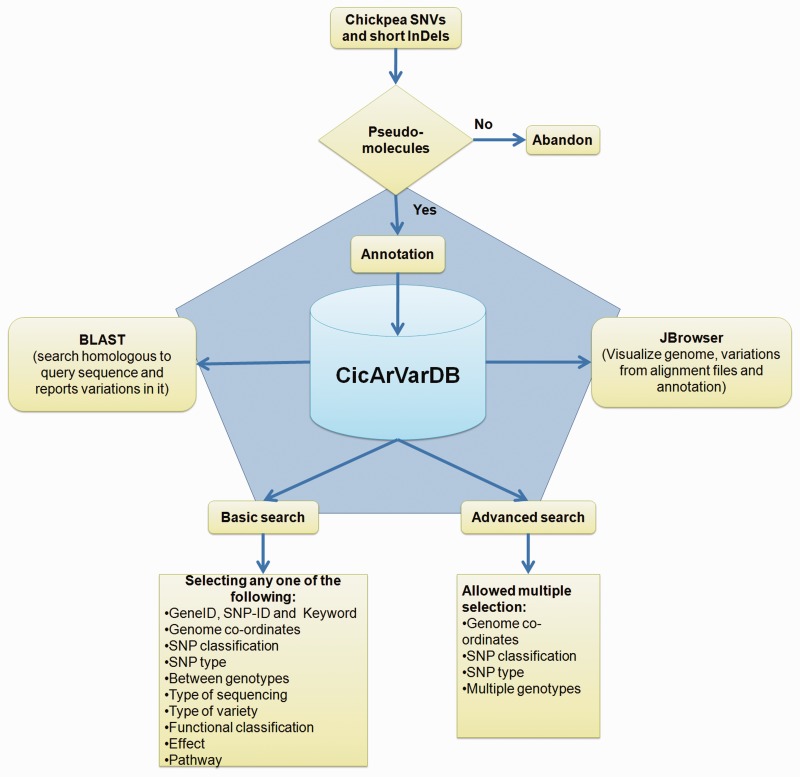
Schematic outline of CicArVarDB.

Genes and gene annotations were downloaded from http://www.icrisat.org/gt-bt/ICGGC/genomedata.zip. Functional annotation for the predicted chickpea gene set was done using BLASTP (25) comparison with the Swiss-Prot and TrEMBL databases with an e-value threshold of 1e−05. Corresponding UniProt IDs were obtained. The annotated genes were classified based on gene-ontology, domain specificity, protein family and the pathways they featured in from UniProt Knowledge Base (UniProtKB) (26) using a custom made perl script (Uniprot_to_functional_annotation.pl; https://github.com/CEG-ICRISAT/cicarvardb-scripts). Processed SNP information along with genes and their annotation were split into five tables. The schematic overview of the CicArVarDB is described in Figure 1.

The web interface was developed using the web-builder platform WordPress. Currently the database, CicArVarDB is hosted on Amazon cloud server with two cores and 8 GB RAM. It is configured with BLAST and JBrowse (27) to enable sequence based searching for SNP containing loci and visualisation of the alignment of reads mapped to the reference genome sequence.

## Results and discussion

From an initial dataset of 4.4 million genomic variants reported by Varshney *et al.* (2), around 2.5 million were removed as they could not be mapped to any of the chickpea pseudomolecules. A final set of 1 965 803 variations, included SNPs (88.48%) and small length (1–10 bp) insertions (5.27%) and deletions (6.23%). InDels of length 1 and 2 bp contributed more than 70% of total small InDels.

**Figure 2.**
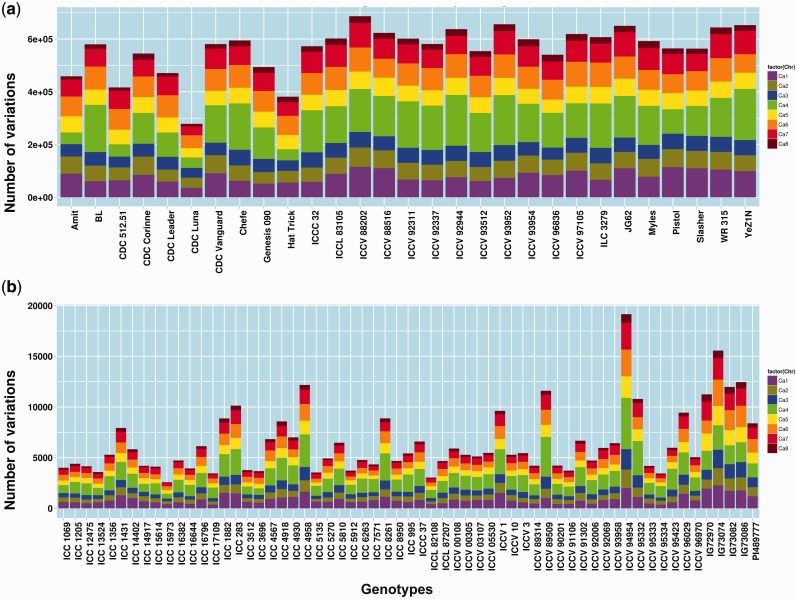
Genome wide distribution of variations across 90 chickpea genotypes. a) distribution of variation among 29 lines re-sequenced using WGRS approach. b) distribution of variations among 61 lines re-sequenced using RAD-Seq approach.

The minimum variations amongst the WGRS lines was found to be 278 530 in CDC Luna, while the maximum variations was 686 712 in ICCV 88202. In the RAD sequenced genotypes, the minimum variations count was 2580 in ICC 15973 and the maximum, 19 160, was reported in ICCV 94954 (Figure 2a,b). Detailed distribution of variations across pseudo-molecules and other features can be referred to in Supplementary Table S1.

**Figure 3.**
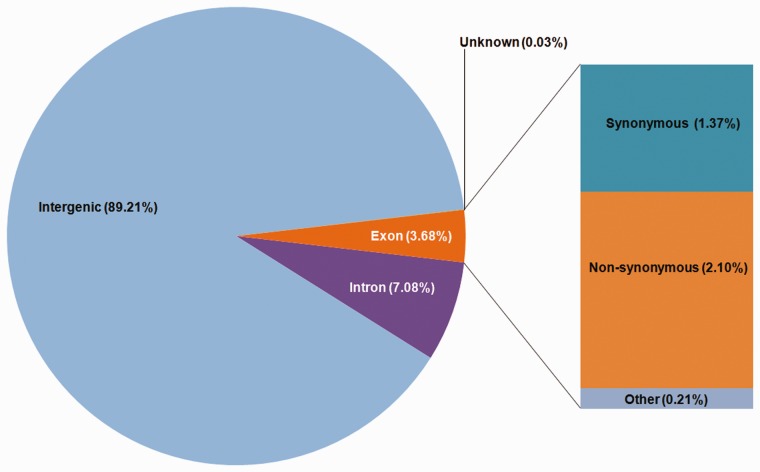
Distribution of variations on the basis of the genomic locations.

Variations were classified on the basis of their genomic locations using SnpEff (28). The most abundant variations (89.21%) were found in intergenic regions with a density of 6.38 variations per kilo base pair while the variation density in exonic and intronic regions were found to be 2.64 (3.68%) and 3.16 (7.08%), respectively (Figure 3; Table 1). Variations within coding regions were further functionally classified into missense (57.94%), nonsense (2.22%) and silent (39.83%) mutations.

**Table 1.** Variation statistics across the chickpea genome

Region	Length	SNPs	Deletions	Insertion	Total	Density (Per Kb)	Change rate
CDS	27 941 821	71 862	1240	692	73 794	2.64	378.65
Intron	45 204 800	119 662	12 677	10 617	142 956	3.16	316.21
Intergenic	274 100 756	1 547 940	108 729	92 384	1 749 053	6.38	156.71
Total	347 247 377	1 739 464	122 646	103 693	1 965 803	5.66	176.64

The overall transitions/transversions ratio (Ts:Tv) was 1.44 which is slightly lower than that observed in maize nuclear SNPs (1.48) and grass chloroplast (1.3) (14, 16, 23, 29–31). Using SnpEff, the impact of variations on the protein coding sequences was classified into low, moderate and high effect variations. The low effect variations (24 303) included synonymous SNPs and the variations observed in splice site region (Sequence variant where a change has occurred either within 1–3 bases of the exon or 3–8 bases of the intron). Moderate effect variations (36 087) included the ones which change coding region by insertion, deletion or alteration of the codon. Variations that lead to loss of exon, changes in splice site acceptor (two bases before exon start, excluding the first exon) or donor (two bases after coding exon end, excluding for the last exon) and frame shift mutations, etc were grouped as high effect variations (2359).

**Figure 4.**
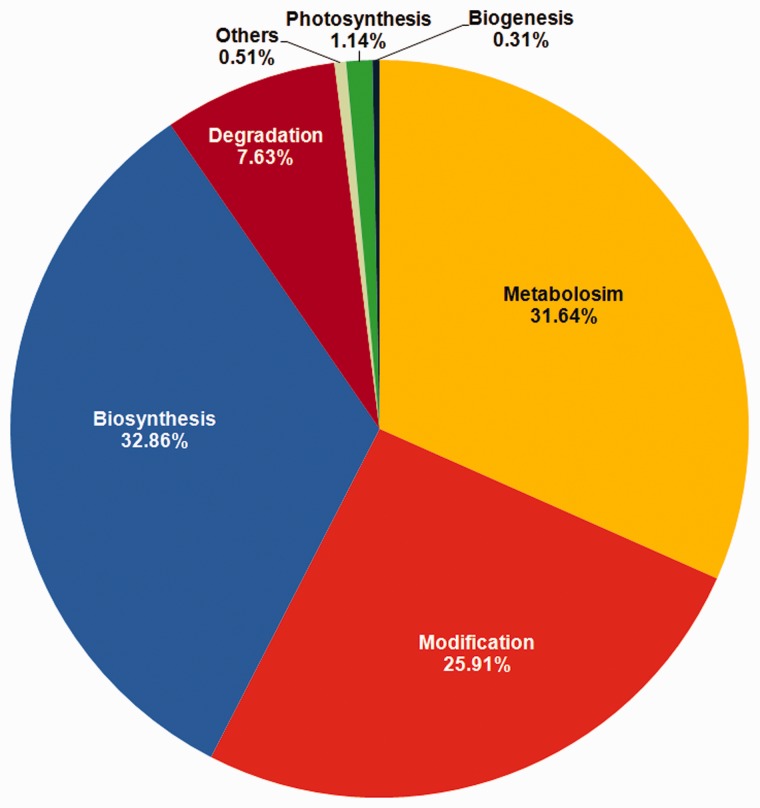
Distribution of variations based on pathway annotation.

Functional annotation of genes containing the variations was carried out by searching for homologous sequences using BLASTP. Genes were grouped into categories including ‘Biogenesis’, ’Biosynthesis’, ’Degradation’, ‘Metabolism’, ‘Modification’ and ’Photosynthesis’. The detailed gene annotation can be obtained by clicking on Gene-ID featuring the corresponding SNP/InDel. Annotation includes UniProt, GO and InterProScan (32) IDs and pathway classification beside other details. User can fetch more details about the genes by clicking on respective UniProt, GO and InterProScan IDs. The maximum number of variations (6128), across 668 genes was observed in the biosynthesis group which included subcategories such as biosynthesis of protein, amino acid, plant hormone etc. The metabolism group contained a total of 672 genes which exhibited 5901 variations followed by Modification group, mainly involved in modification of proteins, containing 741 genes with 4831 variations (Figure 4).

## An overview of CicArVarDB

CicArVarDB is a genome variation mining tool for chickpea and it has two different approaches to screen the variations.

A basic search provides options to narrow down the reported variations. The user has the following options to perform a variations search.

1. **Keyword search:** Variations can be searched based on the gene, SNP-ID or the keywords such as ‘disease resistance’, ‘kinase’, ‘transporter’ etc. Variation search using keywords is supported by boolean operators such as ‘AND’, ‘OR’, ’NOT’ for a combination of words, with space as delimiter, supporting search for the exact string match. The keyword search also supports search for a substring of a given single keyword (no spaces). The search output includes the location of variations if present in the gene of interest.
2. **Location:** Users can search for the presence of variations within specified genome co-ordinates.
3. **SNP classification:** Variations are classified according to their location in the genome such as intergenic, intronic and CDS. Variations within the CDS regions were further classified into synonymous and non-synonymous. Users can search for variations within these categories.
4. **Type of variants:** Variations were categorized as insertions, deletions and substitutions to facilitate the search of variants of choice.
5. **Between any two genotypes:** Search can be executed for variations between any two genotypes of interest.
6. **Sequencing type:** Currently, the database hosts variations obtained from WGRS and RAD sequencing data.
7. **Type of genotypes:** Sequenced *desi* and *kabuli* chickpea genotypes can be filtered for variants occurring in either of these types.
8. **Functional class:** SNPs classified as missense, nonsense and silent can be retrieved.
9. **Effect intensity:** On the basis of the severity of their effects, variations were differentiated into low, moderate and high effect variations. These variations may occur in the intergenic, intronic or exonic regions in the genome.
10. **Pathway:** Search can be carried out for the variations affecting a gene featured in a specific biological pathway.

An advanced search method provides the user with more comprehensive selection criteria to choose at least any two of the listed options:

1. Genomic location
2. SNP classification
3. SNP type
4. Selecting multiple genotypes

An advanced search can be used to attain very specific and precise number of variations. The results of both basic and advanced searches includes a table of genomic co-ordinates, bases present at the SNP position in reference genome sequence and corresponding variant base call. A click on ‘+’ symbol present at the beginning of each record expands the row with two columns displaying genotype names which match to the reference and variant base, respectively. Variation can be visualized in JBrowse by clicking on the SNP ID provided in the results table. The basic search method numbers 6 and 7 list out the genotypes depending on the sequencing type (WGRS/RAD) and the type (*desi*/*kabuli*) of genotype, respectively. Selecting a particular genotype from this table produces a table detailing the variations present in it.

The user may wish to know the presence of reported variations in a sequence of interest. To perform this, webBLAST has been implemented as an additional tool to help the user find a homologous region in the reference sequence with a submitted query sequence and explore the presence of variations within a 10 kb flanking region. BLASTN search is integrated to find such homologous region in the chickpea genome for the user given query sequence and the co-ordinates for resulting HSPs are used to identify the number of SNPs present in the region. The result page displays the SNP information along with its presence in the gene and associated gene annotation.

JBrowse, a Java based genome browser is embedded in the database. JBrowse uses a client side scripting which makes the browser faster and allows easy scaling of large genome regions unlike other genome browsers like GBrowse (which is implemented by CGI protocols). These features of JBrowse enables the users to upload BAM files as tracks to visualize alignment files faster as compared to the CGI-based genome browsers and provides pictorial representation for the presence of variations. By default JBrowse displays seven genoytpes' alignment files as tracks along with gene annotation track. It contains an option of selecting one or more genotypes. An option is provided for selecting multiple genotypes at once based on the grouping like desi/kabuli/wild or WGRS/RAD sequencing.

## Conclusions

Advances in high throughput sequencing have led to the detection of large number of genome wide SNP markers that are valuable tools in plant genomics. These molecular markers are being utilized extensively in breeding programs in many crops. Chickpea genome sequence provides a comprehensive resource for the mining of SNP markers. CicArVarDB is a comprehensive resource which lists SNPs/InDels reported in 90 chickpea genotypes. Moreover, the variations reported have been classified based on their effects and distribution on genome. The variations containing sequences have also been functionally annotated with UniProtKB and InterProScan. This variation database will support the application of existing genomic resources like several thousand simple sequence repeats (SSRs), several million SNPs, high-density diversity array technology (DArT markers) and Illumina GoldenGate assays, high-density genetic maps, transcriptome assemblies and the draft genome sequence of chickpea (33) to chickpea improvement programmes. CicArVarDB has been designed with an intent to help breeders/researchers mine for SNP markers and is supported by a user friendly web interface. It also implements an easy to use visualization tool JBrowse, which provides a pictorial representation of the variations to the user.

### Future directions

The database will be updated periodically based on the availability of the SNP/InDel datasets for genotypes which will be sequenced in future. SNPs/InDels which will be validated in different studies will also be incorporated in the database. We intend to include more features in the database including conversion of SNPs into putative CAPS markers and designing primers for SNPs. Options will be provided to export the SNPs in file formats accepted by various high-throughput SNP genotyping platforms such as the GoldenGate Genotyping Technology (GGGT; Illumina, San Diego, CA, USA), SNPStream (Beckman Coulter, USA), GeneChip (Affymetrix, USA) and KASPar (KBio science, UK)**.**

### Availability and requirements

CicArVarDB is open access and available at http://cicarvardb.icrisat.org. The web interface and the genome browser work with latest browser versions of Mozilla Firefox (10 and later), Google Chrome (17 and later), Apple Safari (5 and later, 6 is required for BAM, BigWig, VCF + Tabix) and Microsoft Internet Explorer (9 and later, 10 is required for BAM, BigWig, VCF + Tabix).

## Supplementary Data

Supplementary data are available at *Database* Online.

Supplementary Data
