# Supplementary material for: CicArVarDB: SNP and InDel database for advancing genetics research and breeding applications in chickpea
Source: Database (Oxford). 2015 Aug 18;2015:bav078. doi: 10.1093/database/bav078 (PMC4541373; doi:10.1093/database/bav078)
Supplement: Supplementary Data [file supp_2015_bav078_index.html]

Supplementary Data 

# CicArVarDB: SNP and InDel database for advancing genetics research and breeding applications in chickpea

## Supplementary Data

files

- Supplementary Data - xlsx file
